# Magnetic Field Sensor Based on a Tri-Microfiber Coupler Ring in Magnetic Fluid and a Fiber Bragg Grating

**DOI:** 10.3390/s19235100

**Published:** 2019-11-21

**Authors:** Fangfang Wei, Dejun Liu, Arun Kumar Mallik, Gerald Farrel, Qiang Wu, Gang-Ding Peng, Yuliya Semenova

**Affiliations:** 1 Photonics Research Centre, School of Electrical and Electronic Engineering, Technological University Dublin, Kevin St, D08 X622 Dublin, Ireland; arunkumar.mallik@tudublin.ie (A.K.M.); gerald.farrell@tudublin.ie (G.F.); yuliya.semenova@tudublin.ie (Y.S.); 2 College of Optoelectronic Engineering, Shenzhen University, Shenzhen 518060, Guangdong, China; dejun.liu@szu.edu.cn; 3 Department of Mathematics, Physics and Electrical Engineering, Northumbria University, Newcastle Upon Tyne NE1 8ST, UK; qiang.wu@northumbria.ac.uk; 4 Photonics & Optical Communications, School of Electrical Engineering & Telecommunications, University of New South Wales, Sydney 2052, Australia; g.peng@unsw.edu.au

**Keywords:** magnetic field sensor, magnetic fluid, tri-microfiber coupler, taper, fiber Bragg grating (FBG)

## Abstract

In this paper we propose and investigate a novel magnetic field sensor based on a Tri-microfiber coupler combined with magnetic fluid and a fiber Bragg grating (FBG) in a ring. A sensitivity of 1306 pm/mT was experimentally demonstrated in the range of magnetic fields from 0 to 15 mT. The reflection peak in the output spectrum associated with the FBG serves as a reference point allowing to avoid ambiguity in determining the spectral shift induced by the magnetic field. Due to its high sensitivity at low magnetic fields, the proposed structure could be of high interest in low field biosensing applications that involve a magnetic field, such as magnetic manipulation or separation of biomolecules.

## 1. Introduction

Optical fiber interferometers using 2 × 2 couplers, such as Mach–Zehnder, Fabry–Perot or Sagnac loop interferometers have been widely utilized as magnetic field sensors for many applications including navigation, aviation, space and geophysical research, and biosensing [1,2,3]. 

Magnetic sensors based on optical fiber interferometers are an excellent alternative to traditional electronic sensors due to their outstanding advantages of immunity to electromagnetic interference, compact size, and ability to operate in hazardous environments. A number of optical fiber interferometric structures have been proposed for magnetic field sensing, such as fiber gratings [4], microfiber couplers [5,6], surface plasmonic structures [7,8,9], cascaded fiber hetero-structures [10], tapered photonic crystal fibers [11], various fiber structures incorporating magnetic fluids [12], and so on. It should be noted however, that since the main research focus currently is on sensing of the magnetic field strength, most of the existing sensors cannot be used for determining the magnetic field direction. 

Typically, existing fiber-optic sensors suffer from temperature-induced drift and often require additional means for temperature compensation [1,5,7,8,9], and fiber Bragg grating (FBGs) prove to be an effective tool for temperature referencing [4,6]. Finally, many of the fiber-optic sensors incorporating magnetic fluids or nanoparticles suffer from hysteresis due to their inherently slow response times [1,3]. One way to accelerate the response is reduction of nanoparticle concentration in the magnetic fluid, which often leads to a decreased sensitivity to the magnetic fluid.

Traditional fiber interferometers such as Mach–Zehnder, Fabry–Perot, or Sagnac are about ten times more sensitive to environmental parameters compared to modal interferometers [13,14]. One possible way of realizing modal interferometers is by tapering single-mode fibers. Once the radius of the tapered fiber is smaller than the normal fiber core radius, the fundamental core mode (HE11) transitions into higher-order modes (HE11, HE12, HE13...) when entering the first transition region of the taper. The light then propagates within the uniform taper waist region accumulating relative phase differences between the different higher-order modes before they re-couple and interfere again in the second transition region of the taper. The phase difference is vital in interferometer, by inducing birefringence, or change of environmental refractive index (RI) the effective refractive index difference increases [15]. Fiber interferometers employing interference in an optical microfiber coupler fabricated from a single-mode fiber have the advantage of high sensitivity to external environmental parameters due to the larger proportion of evanescent field surrounding the microfiber. In addition, a larger phase difference between the interfering modes can be facilitated by using a polarization controller. Research shows that there are advantages to using 3 × 3 couplers as opposed to 2 × 2 couplers in the Mach–Zehnder and Sagnac sensing configurations, as it provides an instantaneous stable reference arm with low-coherence, allowing for the optical resolution of complex-conjugate ambiguity without phase stepping [16,17,18,19].

In this letter we propose and investigate a novel magnetic field sensor based on a microfiber interferometer combined with a fiber loop containing a 3 × 3 tapered microfiber coupler (Tri-MFC) covered with magnetic fluid (MF) and a fiber Bragg grating (FBG). In addition to sensing of a magnetic field, the FBG could be used for simultaneously providing temperature information. This feature could be particularly useful in many magnetic field sensing applications where there is also a need to measure temperature, for example in research in superconductors. Spectral shift in the output spectrum of the proposed sensor is proportional to the magnetic field strength applied to the Tri-MFC, and the spectral shift of the FBG peak indicates changes in the surrounding temperature.

## 2. Materials and Methods

In the manuscript, the proposed theoretical model based on the coupled-mode theory was analyzed numerically by means of the MATLAB software package (MathWorks). A set of differential equations and the corresponding boundary conditions were derived according to coupled-mode theory.

### 2.1. Materials

The MF sample (IO-A20-5) was employed with 20 nm Fe_4_O_3_ particles at a concentration of 5 mg/mL, which was purchased from Cytodiagnostics Inc. 

### 2.2. Proposed Structure and Fabrication

**Figure 1:**
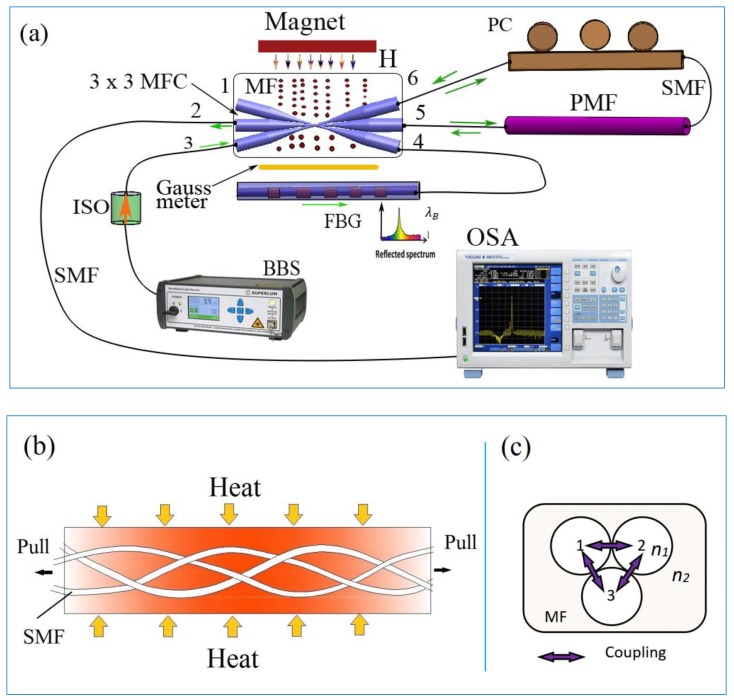
(**a**) Schematic diagram of the proposed sensor; (**b**) Three fibers are slightly twisted together to ensure close proximity among them during the fusing and tapering process. (**c**) Sketch of a cross-section of the 3 × 3 tapered microfiber coupler (Tri-MFC) immersed in magnetic fluid.

A schematic diagram of the proposed sensor is shown in Figure 1a. The structure includes a Tri-MFC (3 × 3 MFC in Figure 1a) made from three single-mode fibers (SMF) fused and tapered together (Figure 1b,c). The Tri-MFC was fabricated using a custom-built fiber tapering setup. Three ~4 cm long sections of a standard single-mode optical fiber (SMF-28, Coring) were stripped of their coating, placed together, and twisted together slightly. Then the coupler was fabricated by simultaneously fusing and tapering the three SMFs using a method known as the microheater brushing technique [20].

Light from a broadband source (BBS) emitting in the wavelength range from 1520 nm to 1580 nm was fed into the MFC input (port 3) and split into beams to ports 4, 5, and 6. Port 4 was connected to the FBG for monitoring of the reflected peak. Port 5 and port 6 were connected by a section of polarization maintaining fiber (PMF) with a ~15 cm length with a polarization controller (PC) included in the loop. The output spectrum of the sensor was monitored at port 2, using an optical spectrum analyzer (OSA) with a resolution of 10 pm. The magnetic field in the experiments was generated by a permanent magnet, with changes in the field strength achieved by changing the distance between the magnet and Tri-MFC immersed in the magnetic fluid (MF).

The schematic of the fusion and tapering process for the Tri-MFC, operating as the sensor’s head, is illustrated in Figure 1b. The waist diameter of each of the microfibers was 2 µm resulting in a total diameter of 6 µm for the Tri-MFC. The uniform taper waist section for the Tri-MFC was about 2.5 cm in length. 

In order to improve the mechanical stability of the MFC in our experiment, polydimethylsiloxane (PDMS) material was used to package the Tri-MFC by encapsulating it in the center of a prefabricated slot with the opposite MFC ends immobilized by a UV curable glue.

The fabricated Tri-MFC was then immersed into magnetic fluid (IO-A20-5, 20 nm particles, 5 mg/mL, from Cytodiagnostics Inc.), of which refractive index (RI) in the absence of magnetic field was 1.350. A sketch of the Tri-MFC immersed into the MF is shown as Figure 1c. Varying magnetic fields in the range from 0 to 15 mT were applied to the sensor using a permanent magnet. 

### 2.3. Theoretical Analysis and Operating Principle

Light propagation in the Tri-MFC can be described using the coupled-mode theory method discussed in [16]. It is well known that in an SMF, the normalized frequency V is the cutoff frequency, and single-mode operation occurs when V is less than or equal to 2.405 [13]. For the fiber taper with a waist diameter of 2 µm, the normalized frequency V is calculated to be 7.35, which means that higher-order modes can propagate in the fiber taper leading to modal interference along the length of the fiber taper.

The coupling coefficients for the Tri-MFC are functions of both the fiber parameters and the Tri-MFC geometry [5]. A 3 × 3 fused coupler can be considered as a triangular cross-section fiber coupler as shown in Figure 1c. Considering the rotational symmetry and orthogonality between normal modes in the Tri-MFC, we limited the study to the case where the three coupling coefficients (k) are identical with the same value of k. With a unit power input to the system from port 3 of the Tri-MFC, after coupling, *P*_4_(z), *P*_5_(z), and *P*_6_(z) were the power amplitudes in the three fibers at ports 4, 5, and 6 as shown in Figure 1a. The power exchange between the three fibers is given by [13,18] as follows:

(1) $$\{\begin{array}{c} \frac{dP_{4}\left(z\right)}{z}+j\beta P_{4}\left(z\right)=-jk\left[P_{5}\left(z\right)+P_{6}\left(z\right)\right]\\ \frac{dP_{5}\left(z\right)}{z}+j\beta P_{5}\left(z\right)=-jk\left[P_{4}\left(z\right)+P_{6}\left(z\right)\right]\\ \frac{dP_{6}\left(z\right)}{z}+j\beta P_{6}\left(z\right)=-jk\left[P_{4}\left(z\right)+P_{5}\left(z\right)\right] \end{array}$$

where β is the propagation constant.

In this case, the characteristic equation is independent of the propagation constant β:

(2) $$=-j\left[\begin{array}{ccc} \beta & k & k\\ k & \beta & k\\ k & k & \beta \end{array}\right]$$

The roots of the characteristic equation are:

(3) $$\lambda_{1,2}=-j\left(\beta -k\right),\;\lambda_{3}=-j\left(\beta +2k\right)$$

with the eigenvector of:

(4) $$\varepsilon_{1}=\left[\begin{array}{c} -1\\ -1\\ 1 \end{array}\right],\varepsilon_{2}=\left[\begin{array}{c} 1\\ 0\\ 1 \end{array}\right],{\;\varepsilon }_{3}=\left[\begin{array}{c} 0\\ 1\\ 1 \end{array}\right]$$

The general solutions are:

(5) $$\begin{array}{c} P_{n}\left(z\right)=e^{-j\left(\beta -k\right)z}c_{1}\varepsilon_{1}+c_{2}e^{-j\left(\beta -k\right)z}\left[\varepsilon_{1}+z\varepsilon_{2}\right]+c_{3}e^{-j\left(\beta +2k\right)z}\varepsilon_{3}\\ P_{n}\left(0\right)=\left[\begin{array}{c} 0\\ 0\\ 1 \end{array}\right],\;\left[\begin{array}{c} 0\\ 0\\ 1 \end{array}\right]=c_{1}\left[\begin{array}{c} -1\\ -1\\ 1 \end{array}\right]+c_{2}\left[\begin{array}{c} 1\\ 0\\ 1 \end{array}\right]+c_{3}\left[\begin{array}{c} 0\\ 1\\ 1 \end{array}\right] \end{array}$$

so $c_{1}=c_{2}=c_{3}=\frac{1}{3}$.

Normalized solutions for the optical power coupled into the Tri-MFC ports are then given as follows:

(6) $$\{\begin{array}{c} P_{4}\left(z\right)=j\frac{1}{3}\left[z-2\sin\left(kz\right)\right]e^{-j\beta z}\\ P_{5}\left(z\right)=-j\frac{2}{3}\left(1+\cos\left(kz\right)\right)\sin\left(kz\right)e^{-j\beta z}\\ P_{6}\left(z\right)=j\frac{1}{3}\left[z+2\left(1-\cos\left(kz\right)\right)\right]\sin\left(kz\right)e^{-j\beta z} \end{array}$$

Based on the proposed sensor structure shown in Figure 1a, the optical power *P*_4_(z) passing through the FBG loop, becomes power *P*_1_(z) [21].

(7) $$P_{FBG}\left(z\right)=tanh^{2}\left(\frac{N\eta \left(V\right)\delta n_{0}}{n}\right)P_{4}\left(z\right)$$

where N is the number of periodic variations of refractive index in FBG, η(V) is the fraction of power in the core, V is the normalized frequency, and $\delta n_{0}$ is the variation in the refraction index.

In the proposed sensor structure, the PMF loop and the FBG loop can be treated as separate light paths. When input light was launched into port 3, it split into three signals and passed through the Tri-MFC toward ports 4, 5, and 6. Under these conditions a Sagnac loop was formed between ports 5 and 6 resulting in light interference in the Tri-MFC. By substituting Equation (6) into Equation (1), $P_{5}\left(z\right)=P_{6}\left(z\right)$. After being combined with the light reflected back from the loop containing the FBG, the resulting transmission observed at port 2 can be expressed as:

(8) $$\left(z\right)=\frac{{\left|P_{out}\right|}^{2}}{{\left|P_{in}\right|}^{2}}=\frac{{\left|P_{5}\left(z\right)\right|}^{2}+{\left|P_{6}\left(z\right)\right|}^{2}+2\left|P_{5}\left(z\right)\right|\cdot \left|P_{6}\left(z\right)\right|cos\left(\varphi \right)cos\left(kzL\right)+k\cdot |P_{FBG}{|}^{2}}{{\left|P_{3}\left(z\right)\right|}^{2}}$$

By ignoring some higher-order terms, T(z) can be expressed as:

(9) $$\begin{array}{c} T\left(z\right)=\frac{4}{9}\left(1+\cos\left(kz\right)\right)\left[z+2\left(1-\cos\left(kz\right)\right)sin^{2}\left(kz\right)\right]\cdot cos\left(\varphi \right)cos\left(kzL\right)+k\\ \cdot \frac{1}{9}tanh^{4}\left(\frac{N\eta \left(V\right)\delta n_{0}}{n}\right){\left[z-2\sin\left(kz\right)\right]}^{2} \end{array}$$

where $\varphi =2\pi l\left(n_{0}-n_{e}\right)/\lambda$ is the phase delay accumulated within the PMF loop, θ is the total angle between light polarizations at both ends of the PMF, *l* is the length of PMF, (*n*_o_ − *n*_e_) is the birefringence of the PMF, λ is the operating wavelength, and k is the coupling coefficient of the fiber coupler. L is the length of the tapered waist of the Tri-MFC.

As one can see from Equation (8), the interference of the tapered 3 × 3 coupler is highly affected by the coupling coefficient between the two fiber loops, which has been proven to be wavelength and RI dependent [5], thus making the resulting output spectrum strongly dependent on the coupler’s surrounding environment.

The operating principle of the PMF loop is based on the interference between the clockwise and counterclockwise propagating beams with a π phase difference after passing the length of the PMF. Both loops’ spectra were overlapped at port 2 and observed using an OSA as illustrated in Figure 1a.

## 3. Results and Discussion

**Figure 2:**
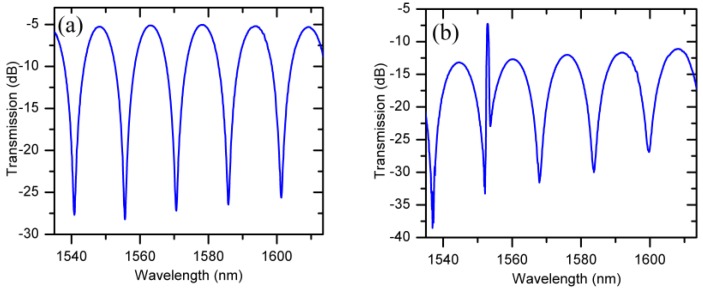
(**a**) Interference spectrum of polarization maintaining fiber - microfiber coupler (PMF-MFC) loop measured at port 2 without the fiber Bragg grating (FBG); (**b**) Combined spectra of the FBG and the PMF-MFC loop measured at port 2.

Figure 2 shows a typical combined spectrum of the PMF loop and the FBG measured at port 2.

In the experiments we used a 15 cm long PMF which resulted in a spectrum with a free spectral range (FSR) of 16 nm. The spectral position of the FBG peak in such a configuration is determined by the temperature only and does not change under the influence of the magnetic field (change of the magnetic fluid RI). However, by measuring the spectral distance between the FBG reflection peak and the nearest selected interference dip, the change in RI (magnetic field) is easily determined, and further, the strength of the applied magnetic field can be measured assuming a suitable calibration has taken place. The experimentally measured transmission spectrum of the FBG and PMF loops recorded at port 2 is shown in Figure 2a.

**Figure 3:**
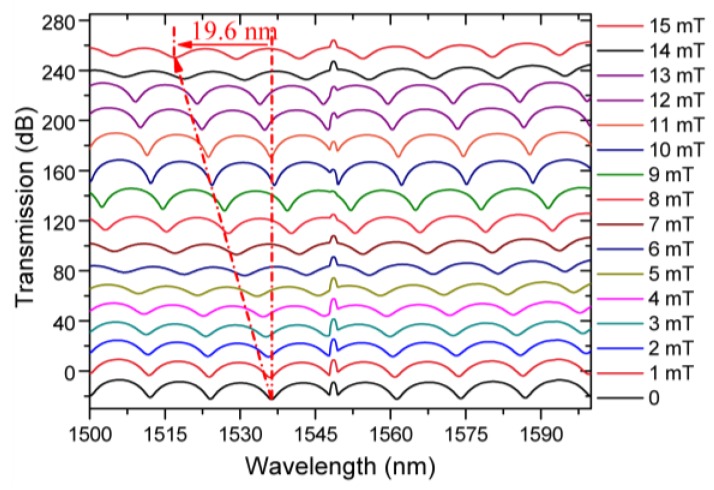
Experimental output spectra for the sensing head immersed in a MF containing 12 nm-diameter Fe_4_O_3_ particles with concentration of 5 mg/mol at different magnetic field strengths ranging from 0 to 15 mT.

Figure 3 shows experimentally measured transmission spectra of the proposed sensor under different magnetic field strengths. An increase in the magnetic field strength applied to the sensor head (Tri-MFC immersed in the magnetic fluid) from 0 to 15 mT results in a blue spectral shift of the selected interference dip by 19.6 nm. Thanks to the presence of the FBG peak as a reference point in the output spectrum, it is possible to avoid ambiguity in determining the shift of the interference spectrum. For example, if the FBG reflection was not present, a 10 mT induced shift of the dip at 1536.3 nm would not be distinguishable from the zero-field neighboring interference dip.

**Figure 4:**
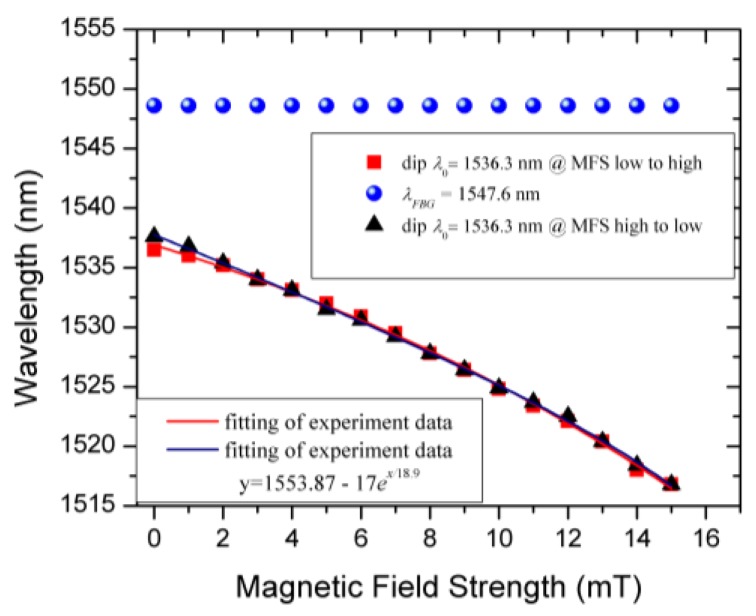
Measured wavelength shift of the selected transmission dip (at 1536.3 nm) and the FBG reflection peak (1547.6 nm) against increasing magnetic field from 0 to 15 mT and then decreased back to zero.

Figure 4 is a plot of spectral positions of the selected transmission dip (near 1536.3 nm) and the FBG reflection peak (1547.6 nm) against the applied magnetic field strength. As can be seen from the plot, the selected transmission dip shifts rapidly from 1536.3 nm to 1516.7 nm within the studied range while the FBG peak remains unaffected.

Figure 4 also illustrates the hysteresis behavior (black and red lines) of the sensor unavoidable due to the limited time necessary for the magnetic nanoparticles to reach a balance after a change in the magnetic field strength or direction. As one can see from the graph, there was a circa 0.1 nm difference between zero-field spectral positions of the selected interference dip after the magnetic field strength was at first increased from 0 to 15 mT, and then decreased back to 0 mT again. It should be noted that the selected spectral dip returned to its initial position approximately 20 min after the magnetic field was reduced to zero.

**Figure 5:**
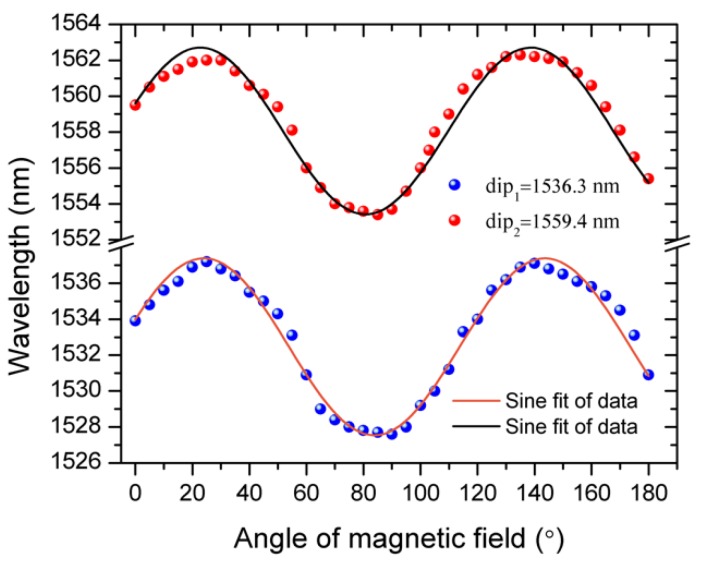
Periodic change in the position of spectral dips with initial positions at 1536.3 nm and 1559 nm when the applied magnetic field direction changes from 0 two 180 degrees.

Figure 5 illustrates the dependences of spectral positions of two selected interference dips on the orientation of the applied magnetic field with an amplitude of 10 mT with respect to the Tri-MFC axis. As one can see from the plot, the dependences are periodic with the change of the direction of the magnetic field from 0 to 180 degrees, demonstrating the potential capability of the proposed sensor to detect field orientation.

**Figure 6:**
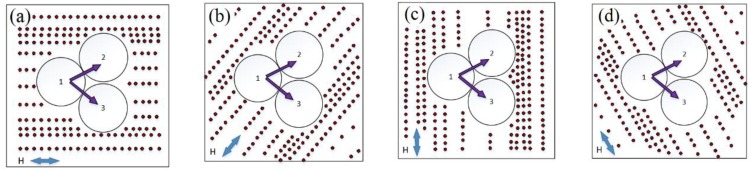
(**a**) External magnetic field direction is at 0° with respect to horizontal axis; (**b**) at 60° with respect to horizontal; (**c**) external magnetic field is vertical (90°); (**d**) external magnetic field direction creates 120° angle with the horizontal axis.

The sensing mechanism of the proposed structure for sensing the orientation of the magnetic field is the orientation-dependent distribution of nanoparticles, leading to the field dependent RI of the magnetic fluid. The distribution of nanoparticles will be influenced by the external magnetic field orientation with respect to the Tri-microfiber coupler (Tri-MFC), whose cross-section at the waist has a triangular symmetry. Figure 6a–d shows that as the external magnetic field direction changes, the magnetic nanoparticles aggregate depending on the field orientation. As was shown in previous reports [5,8], the nanoparticle chains re-align along the magnetic field direction, causing the change of the RI of the surrounding the Tri-MFC fluid.

In Figure 5, the period is about 120°, due to the triangular symmetry of the Tri-MFC cross-section. Besides the main impact from the magnetic field strength, the nanoparticles chain distribution is also influenced by the orientation of the magnetic field with respect to the cross-section of Tri-MFC and magnetic field direction. From Figure 6, when magnetic field is 0°, 60°, and 120°, the distribution of nanoparticles create similar patterns, resulting in periodic changes of the spectral dips positions every 120°. It should be noted that a change in the magnetic field direction from that within the range from 0° to ~ 60° to a value from 60° to ~120°, results in the re-alignment of the nanoparticle chains reversing their collective magnetic moment. Thus, the maximum and minimum spectral dip positions are located at ~30° and 90° in Figure 5.

The effect of temperature has been studied and reported previously [6] for a similar sensing structure containing the same FBG grating as a temperature sensor. Due to this, the results of temperature characterization have not been presented here. It should be noted however that monitoring of the FBG peak spectral position within the proposed structure can be used to provide the surrounding temperature information.

Table 1 compares properties of related fiber-optic magnetic field sensing structures, illustrating that the proposed sensor is capable of sensing magnetic field strength with relatively high sensitivity, and in addition is capable of providing information about magnetic field direction and temperature.

## 4. Conclusions

In conclusion, we have proposed and demonstrated a novel Tri-MFC sensor structure based on a combination of PMF and FBG loops. The sensitivity to magnetic field strength for the proposed structure is 1306 pm/mT for low magnetic fields of up to 15 mT, which is experimentally verified as 2.6 times higher than that for a 2 × 2 MFC based sensor with similar Sagnac structure [5]. Initial results of studies of the influence of the magnetic field orientation on the sensor response indicate the potential capability of the proposed sensor to measure magnetic field direction. The sensor’s high sensitivity in the low fields makes it very promising for biosensing applications.

## Figures and Tables

**Table 1:** Comparison of properties of fiber-optic magnetic sensors reported in the literature.

Scheme	Sensitivity	Vector	Temperature	Reference
Thin-core fiber-FBG	−0.78 dB/m	No	Yes	[4]
2 × 2 MFC Sagnac	−488 pm/mT	Yes	No	[5]
MFC and FBG based fiber laser	102 pm/mT	No	Yes	[6]
Surface Plasmon Resonance (SPR)	10 nm/mT	No	No	[7]
SPR	597.8 pm/Oe	Yes	No	[8]
SPR	0.692 nm/Oe	Yes	No	[9]
Cascaded fiber hetero structures	65.9 pm/Oe	No	Yes	[10]
Photonic crystal fiber	384 pm/Oe	No	No	[11]
Microfiber Knot Resonator	277 pm/mT	No	No	[12]
3 × 3 MFC Sagnac	1306 pm/mT	Yes	Yes	This work
